# Association between the DNA methylations of POMC, MC4R, and HNF4A and metabolic profiles in the blood of children aged 7–9 years

**DOI:** 10.1186/s12887-018-1104-0

**Published:** 2018-03-29

**Authors:** Eun Jin Kwon, Young-Ah You, Bohyun Park, Eun Hee Ha, Hae Soon Kim, Hyesook Park, Young Ju Kim

**Affiliations:** 1 Department of Obstetrics and Gynecology and Ewha Medical Research Institute, Ewha Womans University Medical School, Seoul, 07985 South Korea; 2 Department of Preventive Medicine, Ewha Womans University Medical School, Seoul, 07985 South Korea; 3 Department of Occupational and Environmental Medicine, Ewha Womans University Medical School, Seoul, 07985 South Korea; 4 Department of Pediatrics, Ewha Womans University Medical School, Seoul, 07985 South Korea; 5 Department of Obstetrics and Gynecology, Ewha Womans University Medical School, Seoul, 07985 South Korea

**Keywords:** POMC, MC4R, HNF4A, DNA methylation, Metabolic profiles, Children

## Abstract

**Background:**

Proopiomelanocortin (POMC), melanocortin 4 receptor (MC4R), and hepatocyte nuclear factor 4 alpha (HNF4A) are closely associated with weight gain and metabolic traits. In a previous study, we demonstrated associations between the methylations of POMC, MC4R, and HNF4A and metabolic profiles at birth. However, little is known about these associations in obese children. To evaluate the clinical utility of epigenetic biomarkers, we investigated to determine whether an association exists between the methylations of POMC, MC4R, and HNF4A and metabolic profiles in blood of normal weight and overweight and obese children.

**Methods:**

We selected 79 normal weight children and 41 overweight and obese children aged 7–9 years in the Ewha Birth and Growth Cohort study. POMC methylation levels at exon 3, and MC4R and HNF4A methylation levels in promoter regions were measured by pyrosequencing. Serum glucose, total cholesterol (TC), triglyceride, high-density lipoprotein cholesterol (HDL–c), and insulin levels were analyzed using a biochemical analyzer and an immunoradiometric assay. Partial correlation and multiple regression analysis were used to assess relationships between POMC, MC4R, and HNF4A methylation levels and metabolic profiles.

**Results:**

Significant correlations were found between POMC methylation and HDL–c levels, and between HNF4A methylation and both TC and HDL–c levels. Interestingly, associations were found between POMC methylation status and HDL–c levels, and between HNF4A methylation status and TC levels independent of body mass index.

**Conclusions:**

These findings show that POMC, MC4R, and HNF4A methylation status in the blood of children are associated with metabolic profiles. Therefore, we suggest that the DNA methylation status might serve as a potential epigenetic biomarkers of metabolic syndrome.

## Background

The prevalence of obesity and its related disorders have been increasing worldwide over the last few decades [1]. In particular, the rates of childhood obesity has increased dramatically in many countries, including South Korea [2, 3]. It has been noted that around 40% of overweight children continue to be overweight during adolescence and that up to 80% of obese adolescents become obese adults [4]. Obesity in childhood can therefore severely impact future health outcomes [5].

Obesity is also an important risk factor for the developments of type 2 diabetes, metabolic syndrome (MetS), and cardiovascular disease [6]. Although, the molecular mechanisms underlying the pathogenesis of obesity remain largely unknown, one study concluded that heritability could account for 85% of obesity [7], whereas in another it was estimated heredity accounted for < 2% [8]. The diversity of these findings suggests that obesity is the result of complex interactions between genetic and environment factors.

Epigenetic modifications, especially DNA methylation, are associated with changes of gene expression through binding of transcription factor [9]. The establishment and maintenance of methylation are known to be sensitive to various environmental factors and to be heritable, although the mechanism underlying this inheritance in mammals remains unclear [10]. In rats, maternal diet during pregnancy has been shown to alter DNA methylation patterns and to affect phenotypic consequences [11]. Similarly, human studies have shown that prenatal exposure to famine led to changes in the methylation patterns of genes involved in lipid metabolism [12].

We previously reported that the methylation levels of proopiomelanocortin (POMC), melanocortin 4 receptor (MC4R), and hepatocyte nuclear factor 4 alpha (HNF4A) in cord blood were associated with metabolic profiles [13, 14]. POMC and MC4R genes play critical roles in the regulation of appetite, body weight, and energy homeostasis [15]. The disruptions of POMC or MC4R in humans have been linked to early onset obesity [16, 17]. POMC is cleaved into α-melanocortin stimulating hormones that acts on MC4R in the hypothalamus [17]. MC4R deficiency has been shown to be associated with elevated insulin levels and accelerated linear growth in childhood [17]. HNF4A encodes a transcription factor relevant to glucose and lipid metabolism and has two distinct promoters, P1 and P2, which are separated by more than 45.6 kb [18, 19]. Furthermore, HNF4A mutation has been reported to be associated with impaired insulin secretion, resulting in maturity-onset diabetes of the young [20], and the expression of genes involved in liver and beta-cell functions, such as, glucose transport and glycolysis [20]. To date, most studies have focused on the associations between genetic variants of POMC, MC4R, and HNF4A and metabolic phenotypes [20–22].

In the present study, we analyzed methylation levels of POMC, MC4R, and HNF4A in the blood of normal weight and overweight and obese children aged 7–9 years. We also evaluated associations between these methylation status and metabolic profiles.

## Methods

### Study design

This study was performed on 79 normal weight and 41 overweight and obese children aged 7–9 years that were selected in the Ewha Birth and Growth Cohort study. Methodological details of the cohort study have been reported elsewhere [13, 23]. We choose those with an age- and gender-matched body mass index (BMI) ≥ 85th percentile as overweight and obese children, and those with BMI < 85th percentile as normal weight children based on the 2007 Korean Children and Adolescent Growth Standards [24]. Written informed consent was obtained from parents or guardians with respect to participation in the study. The study was approved by the Institutional Review Board of the Ewha Womans University Hospital (ECT 13-01A-13).

### Anthropometric measurements

Anthropometric measurements and blood sampling were carried out according to the standard procedures by trained researchers. Height and weight were measured to the nearest 0.1 cm and 0.1 kg using an automatic electronic scale (Dong Sahn Jenix Co. Ltd., Seoul, Korea). BMI was calculated by dividing weight by height squared (kg/m^2^).

### Biochemical assessments

Fasting blood samples were collected in 10 ml Vacutainer tubes containing EDTA or serum tubes (BD Biosciences, San Jose, CA). Blood samples were centrifuged at 3000 rpm for 10 min, and the serum so obtained was stored at − 80 °C until required for chemical analysis. Serum glucose, triglyceride (TG), total cholesterol (TC), and high-density lipoprotein cholesterol (HDL-c) were analyzed by an automatic analyzer (model 7180; Hitachi, Tokyo, Japan). Insulin levels were measured using an immunoradiometric assay kit (MyBiosource, San Diego, CA). Insulin resistance was determined by the commonly used homeostasis model assessment of insulin resistance (HOMA-IR) method, which was calculated as (plasma glucose [mmol/L] × insulin [μIU/mL]) /22.5.

### DNA methylation analysis by pyrosequencing

**Fig. 1:**
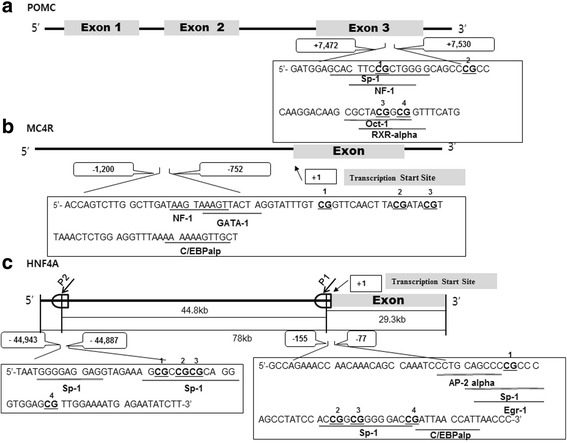
Schematic representation of proopiomelanocortin (POMC) (**a**), melanocortin 4 receptor (MC4R) (**b**), and hepatocyte nuclear factor 4 alpha (HNF4A) (**c**) genes at individual CpG site

Genomic DNA samples were extracted from blood samples of children using the DNeasy Blood and Tissue kit (Qiagen, Hilden, Germany), according to manufacturer’s protocols. Concentrations and purities of DNA were measured using a Nanodrop spectrophotometer (Nanodrop Technologies, Wilmington, DE). Methylation target regions of the POMC, MC4R, and HNF4A were amplified by primer sets designed using the PSQ Assay Design software (Biotage AB, Uppsala, Sweden) [13, 14]. In POMC, the 4 CpG sites are located in the exon 3 region up to 7485 to 7520 bp downstream of the transcription start site (TSS) (Fig. 1a, 13). In MC4R, the 3 CpG sites are located in the promoter region up to − 800 to − 783 bp upstream of the TSS (Fig. 1b, 14). The HNF4A gene has 2 promoters (P1 and P2). The 4 CpG sites in P1 promoter region are located up to − 119 to − 92 bp and 4 CpG sites in P2 promoter region are located up to − 44,924 to − 44,907 bp upstream of the TSS (Fig. 1c, 14). Of these, the CpG2 site in P2 promoter of HNF4A was almost fully methylated (> 99%), and thus, was excluded from this study. Details of pyrosequencing analysis have been described in our previous study [13, 14]. The PCR conditions were as follows; 94 °C for 10 min, followed by 45 amplification cycles (denaturation 94 °C for 30 s, annealing for 30 s, and extension 72 °C for 30 s). For the annealing step, the temperature were set at 52, 53, 54, or 56 °C for POMC, MC4R, HNF4A-P1, and HNF4A-P2, respectively. After amplification, all reaction were incubated at 72 °C for 10 min and then cooled to 4 °C.

### Statistical analysis

Quantitative data were expressed as means ± standard deviations and were analyzed using the Student t-test or analysis of covariance, adjusted for age. TG and insulin levels were analyzed as log-transformed values to satisfy normality. Partial correlation was used to identify the relationship between POMC, MC4R, and HNF4A methylation levels and metabolic profiles after adjusting for age in all subjects. To control for the effects of BMI as a major risk factor of MetS, multiple linear regression analysis was used to assess the associations between DNA methylations of the three genes and metabolic profiles in all subjects. In addition, age and gender were adjusted in the analysis. The statistical analysis was conducted using SAS software (ver. 9.4; SAS Institute Inc., Cary, NC). All analyses were two-tailed, and *p* values of < 0.05 were considered as significant.

## Results

### General characteristics of the study subjects

Seventy-nine normal weight and 41overweight and obese children were included in this study (Table 1). There were significant differences between the two groups in age, height, weight, BMI, and waist circumference (*p* <  0.001). After adjusting for age, average TG, TC, insulin, glucose levels and HOMA index were significantly higher in overweight and obese children (*p* <  0.05), whereas mean HDL-c level was significantly lower (*p* <  0.001).

**Table 1:** General characteristics of the normal weight and overweight and obese children

Characteristics	Normal weight children (*n* = 79)	Overweight and obese children (*n* = 41)	
	Mean ± SD or N (%)	Mean ± SD or N (%)	*p*
Children features			
Age (years)	7.03 ± 0.16	8.24 ± 0.83	< 0.001
Gender (male, n)	39 (49.4%)	21 (51.2%)	0.85
Height (cm)	123.98 ± 5.46	134.06 ± 6.68	< 0.001
Weight (kg)	24.14 ± 3.94	38.32 ± 7.03	< 0.001
BMI(kg/m^2^)	15.61 ± 1.22	21.47 ± 1.83	< 0.001
Waist circumference(cm)	53.56 ± 3.98	69.84 ± 7.55	< 0.001
Blood metabolic profiles^*, ‡^			
TG (mg/dL) ^a^	58.26 ± 1.61	79.18 ± 1.53	< 0.001
TC (mg/dL)	159.56 ± 24.93	165.22 ± 19.34	< 0.001
HDL-c (mg/dL)	61.89 ± 12.00	56.22 ± 9.94	< 0.001
Insulin (μU/mL) ^a^	6.96 ± 1.27	10.80 ± 1.39	0.03
Glucose (mg/dL)	77.94 ± 6.35	78.22 ± 5.72	< 0.001
HOMA^‡^	1.36 ± 0.42	2.22 ± 0.92	< 0.001

*p* values in children features were calculated using the Student *t*-test and ^‡^*p* values in metabolic profiles were calculated using ANCOVA adjusted for age*

^a^TG and insulin levels were analyzed as log-transformed values and results are presented as back-transformed means

*TG* triglyceride, *TC* total cholesterol, *HDL–c* high-density lipoprotein cholesterol, *HOMA* homeostasis model assessment

### Average DNA methylation levels

The following methylation levels were significantly less methylated in overweight and obese children (*p* <  0.05): POMC methylation at 4 CpG sites, MC4R methylation at CpG3, and HNF4A methylation levels at CpG2, 3, and 4 in P1. On the contrary, the following methylation levels were significantly more methylated in overweight and obese children (*p* <  0.05): MC4R methylation at CpG1 and 2, and HNF4A methylation at CpG1 in P1 and at CpG1 and 3 in P2, after adjusting for age (Table 2).

**Table 2:** Average methylation levels of POMC, MC4R, and HNF4A in normal weight children and overweight and obese children

CpG methylation^+^	Normal weight children (*n* = 79)	Overweight and obese children (*n* = 41)	
	Mean ± SD	Mean ± SD	*p*
POMC			
POMC–CpG1^a^	57.07 ± 9.75	54.90 ± 8.10	< 0.001
POMC–CpG2^a^	50.30 ± 9.58	49.07 ± 7.41	< 0.001
POMC–CpG3^a^	51.71 ± 10.25	50.42 ± 7.46	0.002
POMC–CpG4^a^	50.83 ± 9.15	49.20 ± 7.05	< 0.001
MC4R			
MC4R–CpG1	95.19 ± 3.00	95.49 ± 2.51	0.02
MC4R–CpG2	91.03 ± 1.33	91.05 ± 1.61	< 0.001
MC4R–CpG3^a^	83.20 ± 1.71	83.18 ± 1.31	0.01
HNF4A–P1			
HNF4A–CpG1	91.62 ± 4.68	91.73 ± 3.74	< 0.001
HNF4A–CpG2^a^	80.64 ± 6.30	80.53 ± 4.25	0.01
HNF4A–CpG3^a^	85.83 ± 2.96	85.32 ± 2.37	0.001
HNF4A–CpG4^a^	86.24 ± 2.79	85.18 ± 2.99	0.02
HNF4A–P2			
HNF4A–CpG1	96.93 ± 1.07	97.55 ± 0.91	0.01
HNF4A–CpG3	94.79 ± 1.28	95.40 ± 1.05	0.03
HNF4A–CpG4^a^	91.44 ± 1.84	91.29 ± 1.80	0.05

^+^*P* values were calculated using ANCOVA adjusting for age

^a^indicate lower methylation

*POMC* proopiomelanocortin, *MC4R* melanocortin 4 receptor, *HNF4A* hepatocyte nuclear 4 alpha, *P* promoter region

### The relationship between DNA methylation and blood metabolic profiles

Table 3 shows significant correlations between hypermethylation of POMC at 4 CpG sites, and between hypermethylation of HNF4A at CpG4 in P1 and lower HDL-c levels (*r* = − 0.19 ~ 0.23, *p* <  0.05). In addition, hypomethylation of HNF4A at CpG4 in P1, and hypermethylation of HNF4A at CpG3 in P2 were significantly correlated with higher TC levels (*r* = − 0.32, *p* = 0.001, *r* = 0.30, *p* = 0.001, respectively). Hence, we focused on these 6 CpG sites and 2 metabolic profiles showing significant relation (*p* <  0.05).

**Table 3:** Partial correlations between the methylations of POMC, MC4R, and HNF4A and metabolic profiles adjusted for age, Unit: r (*P*)

	TG	TC	HDL-c	Insulin	Glucose	HOMA
POMC						
POMC–CpG1	0.03 (0.75)	− 0.04 (0.71)	− 0.20 (0.03)^a^	− 0.03 (0.73)	−0.03 (0.76)	− 0.05 (0.60)
POMC–CpG2	0.04 (0.70)	−0.09 (0.34)	−0.23 (0.01)^a^	− 0.03 (0.75)	−0.05 (0.61)	− 0.06 (0.54)
POMC–CpG3	0.06 (0.53)	−0.04 (0.71)	−0.19 (0.04)^a^	− 0.03 (0.77)	−0.02 (0.84)	− 0.05 (0.62)
POMC–CpG4	0.02 (0.87)	−0.05 (0.56)	−0.20 (0.03)^a^	− 0.06 (0.52)	−0.05 (0.58)	− 0.09 (0.35)
MC4R						
MC4R–CpG1	0.14 (0.14)	−0.03 (0.78)	−0.15 (0.11)	0.14 (0.14)	0.03 (0.79)	0.12 (0.18)
MC4R–CpG2	0.08 (0.41)	−0.03 (0.74)	−0.03 (0.72)	0.18 (0.06)	−0.05 (0.58)	0.12 (0.20)
MC4R–CpG3	0.06 (0.50)	−.0.06 (0.50)	−0.05 (0.61)	−0.14 (0.14)	0.00 (1.00)	−0.13 (0.16)
HNF4A-P1						
HNF4A–CpG1	0.12 (0.21)	−0.003 (0.98)	0.03 (0.72)	0.14 (0.14)	0.06 (0.51)	0.12 (0.20)
HNF4A–CpG2	0.12 (0.20)	−0.13 (0.17)	−0.04 (0.65)	0.07 (0.49)	0.14 (0.14)	0.11 (0.23)
HNF4A–CpG3	−0.12 (0.19)	−0.19 (0.05)	− 0.17 (0.07)	−0.17 (0.08)	0.01 (0.94)	−0.16 (0.09)
HNF4A–CpG4	0.02 (0.80)	−0.32 (0.001)^a^	− 0.20 (0.04)^a^	− 0.10 (0.30)	−0.01 (0.92)	− 0.09 (0.35)
HNF4A-P2						
HNF4A–CpG1	0.003 (0.98)	0.00 (1.00)	0.04 (0.69)	−0.15 (0.10)	−0.09 (0.32)	− 0.11 (0.24)
HNF4A–CpG3	−0.04 (0.64)	0.30 (0.001)^a^	0.11 (0.25)	0.04 (0.69)	−0.02 (0.86)	0.06 (0.55)
HNF4A–CpG4	0.03 (0.77)	−0.07 (0.43)^a^	− 0.14 (0.14)	−0.05 (0.62)	− 0.05 (0.60)	−0.01 (0.88)

^a^indicate statistically significant correlations

*POMC* proopiomelanocortin, *MC4R* melanocortin 4 receptor, *HNF4A* hepatocyte nuclear 4 alpha, *P* promoter region, *TG* triglyceride, *TC* total cholesterol, *HDL–c* high-density lipoprotein cholesterol

### The association between DNA methylation and blood metabolic profiles in all subjects

To control for the effect of BMI on metabolic profiles, we analyzed associations between the methylation status of POMC, MC4R, and HNF4A and metabolic profiles after adjusting for age, gender, and BMI in all subjects (Table 4). We observed a hypermethylation of POMC-CpG2 was significantly related to a lower HDL-c level (β = − 0.23, *p* = 0.048). Hypomethylation of HNF4A-CpG4 in P1 and hypermethylation of HNF4A-CpG3 in P2 were significantly associated with higher TC levels (β = − 2.79, *p* = 0.001; β = 5.70, *p* = 0.002).

**Table 4:** Associations between DNA methylation status at CpG sites and metabolic profiles adjusted for age, gender, and BMI

	TC		HDL–c	
	β (SE)	*p*	β (SE)	*p*
POMC				
POMC–CpG1	−0.15 (0.24)^*^	0.52	−0.20 (0.11)^*^	0.08
POMC–CpG2	−0.32 (0.25)^*^	0.20	−0.23 (0.12)^*,a^	0.048^a^
POMC–CpG3	− 0.16 (0.24)^*^	0.50	− 0.18 (0.11)^*^	0.12
POMC–CpG4	−0.23 (0.26)^*^	0.39	−0.20 (0.12)^*^	0.10
HNF4A–P1				
HNF4A–CpG4	−2.79 (0.76)^*,a^	0.001^†^	−0.67 (0.37)^*^	0.07
HNF4A–P2				
HNF4A–CpG3	5.70 (1.76)^*,a^	0.002^†^	1.14 (0.86)	0.19

Results are presented as coefficients (β) and SE after adjusting for age, gender, and BMI

^a^indicate statistically significant associations. * indicates the hypomethylated

## Discussion

Epigenetic variations in metabolism–related genes partly contributed to the development of obesity and MetS. Most studies have reported the associations between POMC, MC4R, and HNF4A variants and obesity and MetS-related phenotypes [16–20]. In this study, we identified significant correlations between the methylation status of POMC and HNF4A and metabolic profiles in the blood of normal weight and overweight and obese children. Moreover, BMI-independent associations were found between the methylation status of POMC and HNF4A, and TC and HDL–c levels in Korean children. These findings suggest that epigenetic changes in metabolism-related genes may influence metabolic profiles and could serve as potential epigenetic biomarkers for MetS.

In the present study, mean TG, TC, insulin, and glucose levels and HOMA index were significantly higher in overweight and obese children, whereas mean HDL–c levels had significantly lower than in normal weight children. Ba et al. also found that mean TG, TC, and insulin levels and HOMA index were significantly higher in obese children than in normal children [25]. Similarly, Azab et al. reported that obese children had significantly higher TG and TC levels, and lower HDL-c levels than non-obese children [26]. However, Woo et al. reported that mean TC levels were not different between obese and non-obese children, although mean TG and insulin levels were significantly different [27].

Furthermore, our results indicate average POMC, MC4R, and HNF4A methylation levels at CpG sites were significantly different in overweight and obese and normal weight children. Interestingly, our earlier study found that POMC, MC4R, and HNF4A methylation in specific CpG sites were significantly different in term and preterm infants [14].

In this study, the methylation levels at some CpG sites differed by < 1% in the two groups. Although these differences were quite marginal, they were found to be statistically significant. These results may be due to higher rate of overweight and obese children (34.2%) in this study rather than in the general population. A lower rate of overweight and obese children may have reduced the bias and possibly increased the difference in methylation levels between the two groups. After adjusting for age, we found a negative correlation between POMC methylation levels and HDL–c levels in children. In a previous study, we identified that high POMC methylation levels were marginally related to lower HDL–c levels [13]. Although there is no significant association between MC4R methylation and insulin levels in this study, larger sample size and further study are needed to clarify this association. Martinelli et al. reported that MC4R–deficient children had considerably higher insulin level [17]. Furthermore, a study on MC4R–null mice reported elevated insulin levels [28]. In this study, negative correlations were found between HNF4A methylation levels in P1 and TC and HDL–c levels, whereas HNF4A methylation levels in P2 were positively correlated with TC levels. Hayhurst et al. found that HNF4A–null mice showed markedly lower TC and TG levels [29]. In addition, we previously reported significant associations between the hypomethylation status of MC4R and HNF4A and higher TG levels in term infants and preterm infants [14]. These findings suggest that the methylation status of POMC, MC4R, and HNF4A may contribute to the metabolic phenotypes of children.

Interestingly, we observed a significant association between hypermethylation of POMC–CpG2 in exon 3 and lower HDL–c levels after adjusting for age, gender, and BMI. Crujeiras et al. showed hypermethylation of the promoter region of POMC was associated with weight gain after an energy-restricted diet [30]. In addition, hypermethylation of exon 3 of POMC has been shown to be highly related to childhood BMI, and to be a relatively stable region in different cell types [31]. Biological actions of POMC–derived peptides are mediated by melanocortin receptors [17]. Perez-Tilve et al. reported that inhibition of melanocortin signaling led to higher HDL-c levels independent of body weight [32]. In the current study, hypermethylation of the promoter of MC4R-CpG1 tended to be related to higher insulin levels in children, although not statistically significant. In contrast, Widiker et al. found the hypomethylation status of MC4R was related with obesity in mice fed a high–fat diet [33]. Likewise, the hypomethylation of MC4R was found to be significantly linked with elevated TG levels in cord blood of term infants and preterm infants [14]. These observations suggest that at some CpG sites, MC4R methylation patterns in early life may be influenced by environmental factors rather than by heritable traits. Longitudinal studies and larger sample sizes are required to determine whether MC4R methylation status could impair metabolic profiles and possibly increase the risk of MetS. This study demonstrated BMI-independent associations between the methylation status of HNF4A-CpG4 in P1 and of HNF4A-CpG3 in P2, and TC levels. In humans, HNF4A methylation levels have been reported to differ in neonates with intrauterine growth restriction [34]. These findings concur with those of Ribel–Madsen et al. who found HNF4A was hypermethylated in individuals with type 2 diabetes [35].

This study had several limitations. First, we did not examine methylation levels in target organ tissues, such as, hypothalamus or liver. In addition, we did not take into account cellular heterogeneity in blood samples which may influence methylation levels. Because the study was conducted on children, it was difficult to obtain tissues and samples sizes were limited, and thus, we evaluated DNA methylation levels in whole blood, which is a readily accessible for the assessment of biomarkers. Second, we did not evaluate the environment or social-economic factors, which may affect methylation. Nonetheless, it was identified the methylation status of POMC, MC4R and HNF4A were related to metabolic profiles in Korean children. We also confirmed the associations between the methylation status of three genes and metabolic profiles in 1) cord blood of term and preterm infants in a case-control study [14]; 2) cord blood and blood in 90 children aged 7–9 years in a cohort study [13]. Additionally, these methylation status could influence metabolic profiles. Consequently, we suggest that changes in DNA methylation patterns in childhood may increase the risk of MetS, and longitudinal studies with larger sample sizes would be needed to investigate further.

## Conclusions

This study demonstrates the associations between the methylation status of POMC, MC4R, and HNF4A and metabolic profiles in Korean children. Furthermore, we found BMI–independent associations between DNA methylations of three genes and TC and HDL–c levels. These findings indicate the clinical utility as potential epigenetic biomarkers of MetS, and larger cohort studies are required for further evaluation and verification.

## Abbreviations

BMI: Body mass index
HDL-c: High-density lipoprotein cholesterol
HNF4A: Hepatocyte nuclear factor 4 alpha
MC4R: Melanocortin 4 receptor
MetS: Metabolic syndrome
P: Promoter region
POMC: Pro-opiomelanocortin
TC: Total cholesterol
TG: Triglyceride
TSS: Transcription start site
